# Geographic Distribution and Phylogeny of Soricine Shrew-Borne Seewis Virus and Altai Virus in Russia

**DOI:** 10.3390/v13071286

**Published:** 2021-07-01

**Authors:** Liudmila N. Yashina, Sergey A. Abramov, Alexander V. Zhigalin, Natalia A. Smetannikova, Tamara A. Dupal, Anton V. Krivopalov, Fuka Kikuchi, Kae Senoo, Satoru Arai, Tetsuya Mizutani, Motoi Suzuki, Joseph A. Cook, Richard Yanagihara

**Affiliations:** 1 State Research Center of Virology and Biotechnology “Vector”, 630559 Koltsovo, Russia; smetannikova@vector.nsc.ru; 2 Institute of Systematics and Ecology of Animals, 630091 Novosibirsk, Russia; terio@eco.nsc.ru (S.A.A.); dupalgf54@gmail.com (T.A.D.); krivopalov@gmail.com (A.V.K.); 3 Department of Vertebrate Zoology and Ecology, Tomsk State University, 634050 Tomsk, Russia; alex-zhigalin@mail.ru; 4 Center for Infectious Disease Epidemiology and Prevention Research, Tokyo University of Agriculture and Technology, Tokyo 183-8538, Japan; S203828z@st.go.tuat.ac.jp (F.K.); tmizutan@cc.tuat.ac.jp (T.M.); 5 Center for Surveillance, Immunization and Epidemiologic Research, National Institute of Infectious Diseases, Tokyo 162-8640, Japan; 2319061@ed.tus.ac.jp (K.S.); arais@nih.go.jp (S.A.); mosuzuki@niid.go.jp (M.S.); 6 Faculty of Science, Tokyo University of Science, Tokyo 162-8601, Japan; 7 Department of Biology and Museum of Southwestern Biology, University of New Mexico, Albuquerque, NM 87131, USA; cookjose@unm.edu; 8 Department of Pediatrics, John A. Burns School of Medicine, University of Hawaii at Manoa, Honolulu, HI 96813, USA

**Keywords:** *Hantaviridae*, hantavirus, shrew, evolution, Russia

## Abstract

The discovery of genetically distinct hantaviruses (family *Hantaviridae*) in multiple species of shrews, moles and bats has revealed a complex evolutionary history involving cross-species transmission. Seewis virus (SWSV) is widely distributed throughout the geographic ranges of its soricid hosts, including the Eurasian common shrew (*Sorex araneus*), tundra shrew (*Sorex tundrensis*) and Siberian large-toothed shrew (*Sorex daphaenodon*), suggesting host sharing. In addition, genetic variants of SWSV, previously named Artybash virus (ARTV) and Amga virus, have been detected in the Laxmann’s shrew (*Sorex caecutiens*). Here, we describe the geographic distribution and phylogeny of SWSV and Altai virus (ALTV) in Asian Russia. The complete genomic sequence analysis showed that ALTV, also harbored by the Eurasian common shrew, is a new hantavirus species, distantly related to SWSV. Moreover, Lena River virus (LENV) appears to be a distinct hantavirus species, harbored by Laxmann’s shrews and flat-skulled shrews (*Sorex roboratus*) in Eastern Siberia and far-eastern Russia. Another ALTV-related virus, which is more closely related to Camp Ripley virus from the United States, has been identified in the Eurasian least shrew (*Sorex minutissimus*) from far-eastern Russia. Two highly divergent viruses, ALTV and SWSV co-circulate among common shrews in Western Siberia, while LENV and the ARTV variant of SWSV co-circulate among Laxmann’s shrews in Eastern Siberia and far-eastern Russia. ALTV and ALTV-related viruses appear to belong to the *Mobatvirus* genus, while SWSV is a member of the *Orthohantavirus* genus. These findings suggest that ALTV and ALTV-related hantaviruses might have emerged from ancient cross-species transmission with subsequent diversification within *Sorex* shrews in Eurasia.

## 1. Introduction

Hantaviruses (family *Hantaviridae*) are zoonotic pathogens responsible for causing two febrile syndromes, which are characterized by acute renal failure (known as hemorrhagic fever with renal syndrome) or cardiopulmonary collapse (known as hantavirus cardiopulmonary syndrome) [1,2]. The genome of hantaviruses consists of three negative-polarity, single-stranded RNA segments: small (S), medium (M), and large (L), encoding a nucleocapsid (N) protein, glycoproteins (Gn and Gc), and an RNA-dependent RNA polymerase, respectively. Recently, hantaviruses have been reclassified into four genera—*Orthohantavirus*, *Thottimvirus*, *Loanvirus* and *Mobatvirus*—which demonstrate high genetic distances at the nucleotide (nt) and amino acid (aa) levels [3].

With the discovery of highly divergent hantaviruses in shrews, moles and bats [4,5], long-held conjectures of the co-evolution of rodent-borne hantaviruses and their hosts have given way to concepts of a far more complex evolutionary history, punctuated by cross-species transmission and host switching [6,7,8,9,10,11] and reassortment events [12,13,14,15,16,17]. Furthermore, apart from examples of a single hantavirus species being harbored by multiple reservoir host species, certain host species are known to serve as reservoirs of more than one hantavirus species [9,16,18]. Two highly distinct hantaviruses, Bruges (BRGV) and Nova (NVAV), were found co-circulating among European moles (*Talpa europaea*) [16]. Phylogenetic placement of one of these viruses (BRGV) corresponded to the co-evolution hypothesis, while the position of the second hantavirus (NVAV) suggested cross-species transmission and an ancient reassortment event [16].

The first shrew-borne orthohantavirus, Seewis virus (SWSV), originally detected in the Eurasian common shrew (*Sorex araneus*) in Switzerland [19], and subsequently in archival tissues from common shrews captured in Hungary and Finland [20], has now been found across the vast distribution of its host, spanning across Europe and to the Baikal Lake region in Asian Russia [8,21,22]. SWSV has also been detected in other *Sorex* species, including the tundra shrew (*Sorex tundrensis*) [22], Siberian large-toothed shrews (*Sorex daphaenodon*) [22] and Eurasian pygmy shrew (*Sorex minutus*) [23]. In addition to SWSV, we previously identified a novel highly divergent virus, designated Altai virus (ALTV), in a common shrew captured in August 2007 near Teletskoye Lake, Altai Republic, in Russia [22]. Phylogenetic analysis of the partial L-segment sequence of ALTV (GenBank EU424341) indicated that it was more closely related to mobatviruses, associated mainly with bats, and also suggested ancient host-switching events [9]. Closely related virus sequences were detected from *Sorex araneus* in Hungary and Finland [8,9]. At the same time and in the same location, we found ALTV and SWSV within populations of Eurasian common shrews and Artybash virus (ARTV) among Laxmann’s shrews (*Sorex caecutiens*) [9,24]. Currently, ARTV is reclassified as a genetic variant of SWSV [3]. 

Co-circulation of two highly distinctive hantaviruses, Lena River (LENV) and ARTV, was found within populations of *S. caecutiens* in Khabarovsk Krai and Sakha Republic, Russia [9,25]. It was shown that LENV was most closely related to ALTV and considered as a highly divergent genetic variant of ALTV. Findings also suggested species-shift events during evolution of shrew-borne hantaviruses. Here, we report the co-circulation of SWSV and ALTV in the Tomsk region of Western Siberia and demonstrate that ALTV and LENV likely represent new species of the genus *Mobatvirus* in the family *Hantaviridae*. 

## 2. Materials and Methods

### 2.1. Trapping and Sample Collection

During 2018–2019, *Sorex* shrews were captured in Western Siberia. Field procedures and protocols were approved by the Institutional Animal Care and Use Committees of the Institute of Systematics and Ecology of Animals and the University of New Mexico, following guidelines of the American Society of Mammalogists [26,27]. Collection sites in the Altai Republic were located near Teletskoye Lake (51.79424° N/87.30447° E) and the settlement Choya (52.01558° N/86.49619° E), and in the Tomsk Oblast along the Ob River near settlements Volkovo (58.385195° N/E82.90220° E), Parabel (58.69830° N/81.41260° E), Kargasok (59.02631° N/80.73650° E) and Belyi Yar (58.43178° N/84.96320° E). Lung samples were either frozen in liquid nitrogen or stored in RNA*later*™ (Qiagen, Hilden, Germany) before analysis. Additional shrew specimens were collected, as part of the Beringian Coevolution Project [28], along the Amga River, 10 km NE Sulgachi (61.58046° N/133.14386° E), 7 km N Sulgachi (61.59218° N/132.93862° E) and 8 km ENE Mikhaylovka (61.24610° N/132.71483° E); Kenkeme River, 40 km W Yakutsk (62.07003° N/128.93831° E); and Lena River, 2 km NW Tochtur (61.75421° N/129.52548° E), near Yakutsk, the capital of the Sakha Republic in Siberian Russia, during July and August 2006. Descriptions have been provided earlier [9].

### 2.2. RNA Extraction and RT-PCR Analysis

Total RNA was extracted from lung tissues, using the RNeasy MiniKit (Qiagen, Hilden, Germany) or PureLink Micro-to-Midi total RNA purification kit (Invitrogen, San Diego, CA, USA), then reverse transcribed, using the SuperScript III First-Strand Synthesis System (Invitrogen, San Diego, CA, USA) or Expand reverse transcriptase (Roche, Basel, Switzerland) with random hexamers and universal oligonucleotide primer (OSM55, 5′–TAGTAGTAGACTCC–3′), designed from the conserved 3′ end of the S, M and L segments of hantaviruses. For initial screening by nested RT-PCR, previously described genus-specific oligonucleotide primers targeting the partial L-segment sequence were used [9,29]. Oligonucleotide primers (Supplementary Table S1) were designed from consensus regions of other available hantaviruses and combined with the primers that were described previously [20,22,24]. For the amplification of hantavirus genes, a two-step PCR was performed in 20-µL reaction mixtures, containing 250 µM dNTP, 2 mM MgCl_2_, 1 U of HotStart AmpliTaq polymerase (Roche, Basel, Switzerland) or HotStart Taq polymerase (SibEnzyme Ltd, Academtown, Russia) and 0.25 µM of each oligonucleotide primer. Initial denaturation at 94 °C for 5 min was followed by two cycles each of denaturation at 94 °C for 40 s, two-degree step-down annealing from 48 to 38 °C for 40 s, and elongation at 72 °C for 1 min, then 32 cycles of denaturation at 94 °C for 40 s, annealing at 42 °C for 40 s, and elongation at 72 °C for 1 min, in a GeneAmp PCR 9700 thermal cycler (Perkin-Elmer, Waltham, MA, USA). Amplicons were separated by electrophoresis on 1.5% agarose gels and purified using the QIAQuick Gel Extraction Kit (Qiagen, Hilden, Germany). DNA was sequenced directly using an ABI Prism 377XL or ABI Prism 310 Genetic Analyzer (Applied Biosystems, Foster City, CA, USA).

### 2.3. Genetic and Phylogenetic Analysis

Pair-wise alignment and comparison of full-length coding regions of S-, M- and partial L-segment nt and aa sequences of the hantaviruses from *Sorex* shrews, captured in Russia, with representative rodent-, shrew-, mole- and bat-borne hantaviruses were performed, using the ClustalW in BioEdit [30,31,32]. In addition, we reanalyzed the previously reported hantavirus sequences from archival tissues of *S. araneus* shrews captured in Hungary (SWSV MSB94609, SWSV MSB94615, SWSV MSB95361, SWSV MSB95462, SWSV MSB95463, SWSV MSB95464, SWSV MSB95468, SWSV MSB95480) [20], Finland (DGR18207, DGR18228/Etela, DGR18279/Etela, DGR18283 DGR18874/Oulun, DGR18887, DGR18889/Oulun) [20], Russia (SWSV Kemerovo-Sa65, Karasuk-Sa56, Parnaya-Sa1191, Parnaya-Sa1196, Parnaya-Sa1197, Parnaya-Sa1212, Parnaya-Sa1220, Krasn-Sa5, MShush-Sa1077, MShush-Sa1081, Shish-Sa3, Pokrovka-Sa689, SWSV Telet-Sa300, SWSV Telet-Sa321, SWSV Telet-Sa500, Telet-Sa2318), from *S. caecutiens* (ART502, Parnaya-Sc1205, Khekhtsir-Sc1126, Galkino-Sc2712), *S. daphaenodon* (Irkutsk-Sd475) and *S. tundrensis* (Irkutsk-St489, Galkino-St48, Galkino-St2714) captured in Russia [22,24,25,33] and Mongolia (MG373S022, MG361S013), and included the ALTV-like hantavirus sequences amplified from lung tissue of a *S. caecutiens* captured in Krasnoyarsk Krai (Parnaya-Sc1217), as well as the ALTV-like hantavirus full-length genome from *S. caecutiens* in Khabarovsk Krai (Khekhtsir-Sc67) [25] and the ALTV-like hantavirus partial L-segment sequence from a *S. minutus* shrew captured in Chmiel, Poland (Smin1108) [9].

Phylogenetic trees were generated using the Markov chain Monte Carlo (MCMC) methods Mrbayes 3.1.2 [34], under the best-fit general time reversible model of nucleotide evolution with gamma-distributed rate heterogeneity and invariable sites (GTR+I+Γ) [35]. The best-fit model was selected with jModelTest version 2.1.7 [36] for phylogenetic trees. Two replicate Bayesian Metropolis–Hastings MCMC runs, each consisting of six chains of 10 million generations sampled every 100 generations with a burn-in of 25,000 (25%), resulted in 150,000 trees overall.

## 3. Results

### 3.1. Genetic Analysis

**Figure 1:**
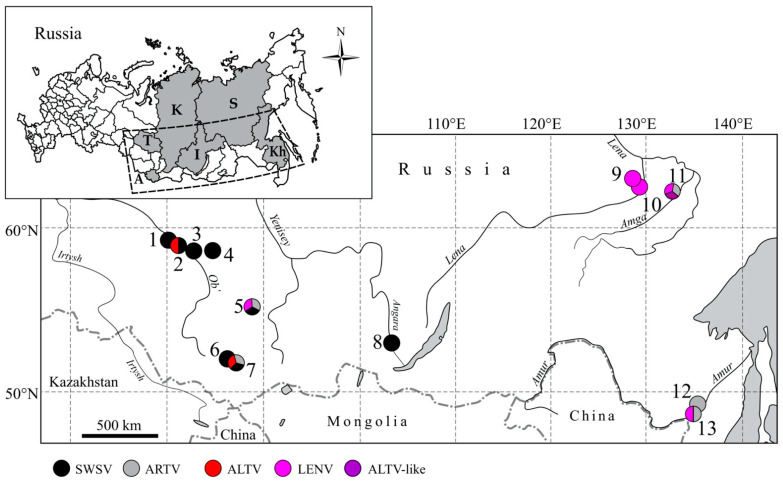
Map, showing the locations of the collection sites in Asian Russia, where hantavirus-infected *Sorex* shrews were captured. (1) Kargasok, (2) Parabel, (3) Volkovo, (4) Belyi Yar, (5) Parnaya, (6) Choya, (7) Teletskoye, (8) Irkutsk City, (9) Kenkeme River, (10) Lena River, (11) Amga River, (12) Galkino and (13) Khekhtsir. The inset shows the geographic locations of (A) Altai Republic, (T) Tomsk Oblast, (K) Krasnoyarsk Krai, (I) Irkutsk Oblast, (Kh) Khabarovsk Krai and (S) Sakha Republic. Samples for the present study are shown in bold type in Table 1. Detected hantaviruses are colored: Seewis virus (SWSV) (black), Artybash virus (ARTV) variant of SWSV (gray), Altai virus (ALTV) (red), Lena River virus (LENV) (magenta), ATLV-like (purple).

During 2018–2019, 173 shrews were captured at two localities of Altai Republic and four localities of Tomsk Oblast, Western Siberia (Table 1 and Figure 1). All samples were analyzed for hantavirus RNA by nested RT-PCR. Hantaviral RNA were identified in 39 *S. araneus* and 1 *S. caecutiens*. The whole genome of ALTV (prototype strain ALT 302), complete S segment sequences (SWSV strain Telet-Sa300, ARTV strain ART 502, LENV strain Parnaya-Sc1217, ALTV strains Parabel-Sa44) were recovered from new and previously reported hantavirus RNA-positive samples (Supplementary Table S2).

Based on the partial L-segment sequences, two distinct *Sorex*-borne hantaviruses were found: strains of both SWSV and ALTV from *S. araneus* (38 and 1 positive/163 tested, respectively) and ARTV variant of SWSV from *S. caecutiens* (1/7) (Table 1, Supplementary Table S2). ALTV and SWSV were found in *S. araneus* at site Parabel in Tomsk Oblast. ARTV in *S. caecutiens* and SWSV in *S. araneus* were detected near Teletskoye Lake, the same site where three *Sorex*-borne hantaviruses, ALTV, SWSV and ARTV, were discovered in 2007. SWSV sequences were recovered from *S. araneus* in the other capture sites. Hantavirus RNA was not detected in *S. isodon* and *S. minutus*, possibly due to the low number of tested samples.

The ALTV virus-positive common shrew sample, designated ALT302, was subjected to full-genome sequencing. The 1987-nt S segment encoded a nucleocapsid (N) protein of 448 aa in length. The 3614-nt M segment contained a single ORF encoding the 1135-aa long glycoprotein precursor (GP) of the Gn and Gc glycoproteins, separated by a WAVSA pentapeptide. The same motif was found in ALTV-related virus from Laxmann’s shrew (Khekhtsir-Sc67) and BRGV from European mole (BE/Vieux-Genappe/TE/2013). The 6533-nt L segment encoded the 2147-aa long RNA-dependent RNA polymerase (RdRp). Analysis of complete S segment-coding sequences demonstrated 12% nt and 2% aa sequence divergence between prototype strain ALT 302 and new geographically distant strain Parabel-Sa44, while showing considerable divergence (>55% nt and >54% aa) between ALTV and SWSV sequences (strain Telet-Sa300), recovered from *S. araneus*, captured in the same site Teletskoye, as well as between ALTV and ARTV (strain ART502). For ALTV strains from Finland (Uurainen63L, LohjaEWS10L), Hungary (MSB95363, MSB95469) and Russia (ALT302, Parabel-Sa44), the divergence of partial S- and L-segment sequences was 18–20% nt (2–3% aa). Analysis of complete S, M, and L sequences revealed more than 23% nt and 15, 14 and 10% aa sequence differences, respectively, between ALT302 and the most closely related ALTV-like virus LENV (strain Khekhtsir-Sc67), and considerable divergence from other representative hantaviruses both at the nt (>27%) and aa (>28%) levels. The observed aa sequence differences exceeded current species demarcation criteria [2], so our data demonstrate that ALTV and ALTV-related virus LENV represent two genetically distinct hantaviruses.

Close identity was detected between LENV strains in *S. caecutiens*, *S. roboratus* and *S. tundrensis*. Complete or nearly complete S-segment sequence divergence between geographically distant strains from *S. caecutiens* (Khekhtsir-Sc67, Parnaya-Sc1217, MSB148793) and *S. roboratus* (MSB148679) and from *S. tundrensis* (MG361S013) located in Russia and Mongolia, respectively, was 11–16% at the nt level and 3–4% at the aa level. 

The new partial L-segment sequences recovered from the remaining 38 hantavirus positive *S. araneus* exhibited 1–12% divergence to each other and to previously published SWSV strains, found in *S. araneus*, *S. tundrensis*, *S. daphaenodon* in Russia, and showed a close evolutionary relationship to those of prototype SWSV mp70 (17–22% nt and 0–2% aa divergence) found in Europe. Partial L-segment sequences, recovered from *S. caecutiens*, captured in the same site Teletskoye in 2007 and 2019 (ART502 and Telet-Sc170) were close to each other (4% nt and 0% aa divergence) and were most closely related with other ARTV variant of SWSV strains from *S. caecutiens* and *S. tundrensis* (Sca383, Khekhtsir-Sc1126, Galkino-St2714 and Galkino-St48) found in distantly located sites in the Sakha Republic and Khabarovsk region (17–20% nt and 0–4% aa divergence). 

### 3.2. Phylogenetic Analysis

**Figure 2:**
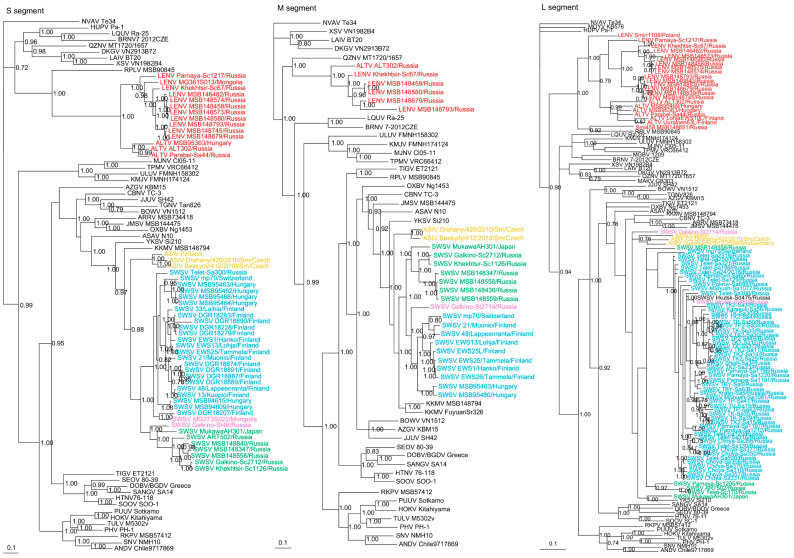
Phylogenetic trees generated by the Bayesian method, under the best-fit GTR+I+Γ model of evolution, based on the S-, M- and L-genomic segments of ALTV (red), SWSV strains from *Sorex araneus* (light blue), SWSV strains from *Sorex caecutiens* (green), SWSV strains from *Sorex tundrensis* (light pink) and Asikkala virus (ASIV) from *Sorex minutus* (yellow). The phylogenetic positions of SWSV, ALTV and LENV strains in Russia are shown in relationship to Thottapalayam thottimvirus (TPMV VRC66412, S: AY526097, M: EU001329, L: EU001330) from *Suncus murinus*, Imjin thottimvirus (MJNV Cl05-11, S: EF641804; M: EF641798; L: EF641806) from *Crocidura lasiura*, Uluguru thottimvirus (ULUV FMNH158302, S: JX193695; M: JX193696; L: JX193697) from *Myosorex geata*, Kilimanjaro thottimvirus (KMJV FMNH174124, S: JX193698; M: JX193699; L: JX193700) from *Myosorex zinki*, Jeju orthohantavirus (JJUV SH42, S: HQ663933; M: HQ663934; L: HQ663935) from *Crocidura shantungensis*, Oxbow orthohantavirus (OXBV Ng1453, S: FJ5339166; M: FJ539167; L: FJ593497) from *Neurotrichus gibbsii*, Cao Bằng orthohantavirus (CBNV TC-3, S: EF543524; M: EF543526; L: EF543525) from *Anourosorex squamipes*, Azagny orthohantavirus (AZGV KBM15, S: JF276226; M: JF276227; L: JF276228) from *Crocidura obscurior*, Bowé orthohantavirus (BOWV VN1512, S: KC631782; M: KC631783; L: KC631784) from *Crocidura douceti*, prototype Seewis orthohantavirus (SWSV mp70, S: EF636024; M: EF636025; L: EF636026) from *Sorex araneus*, Jemez Springs orthohantavirus (JMSV MSB144475, S: FJ593499; M: FJ593500; L: FJ593501) from *Sorex monticolus*, Asama orthohantavirus (ASAV N10, S: EU929072; M: EU929075; L: EU929078) from *Urotrichus talpoides*, Nova mobatvirus (NVA Te34, S: KR072621, M: KR072622, L: KR072623) from *Talpa europaea*, Rockport orthohantavirus (RKPV MSB57412, S: HM015223; M: HM015222; L: HM015221) from *Scalopus aquaticus*, Camp Ripley virus (RPLV MSB90845, S: KF958464, L: KF958465) from *Blarina brevicauda*, Tanganya virus (TGNV Tan826, S: EF050455; L: EF050454) from *Crocidura theresea*, Ash River virus (ARRV MSB73418, S: EF650086; L: EF619961) from *Sorex cinereus*, Yakeshi virus (YKSV Si-210, S: JX465423; M: JX465403; L: JX465389) from *Sorex isodon*, Kenkeme virus (KKMV MSB148794, S: GQ306148; M: GQ306149; L: GQ306150 and KKMV Fuyuan Sr326, S: NC_034559; M: KJ857337; L: KJ857320) from *Sorex roboratus*, and Tigray virus (TIGV ET2121, S: KU934010; M: KU934009; L: KU934008) from *Stenocephalemys albipes*. Shown as well are representative rodent-borne hantaviruses, including Sin Nombre orthohantavirus (SNV NMH10, S: NC_005216; M: NC_005215; L: NC_005217), Andes orthohantavirus (ANDV Chile9717869, S: AF291702; M: AF291703; L: AF291704), Prospect Hill orthohantavirus (PHV PH-1, S: Z49098; M: X55129; L: EF646763), Tula orthohantavirus (TULV M5302v, S: NC_005227; M: NC_005228; L: NC_005226), Puumala orthohantavirus (PUUV Sotkamo, S: NC_005224; M: NC_005223; L: NC_005225), Sangassou orthohantavirus (SANGV SA14, S: JQ082300; M: JQ082301; L: JQ082302), Soochong orthohantavirus (SOOV SOO-1, S: AY675349; M: AY675353; L: DQ056292), Hokkaido virus (HOKV Kitahiyama, S: AB675463; M: AB676848; L: AB712372), Dobrava/Belgrade orthohantavirus (DOBV/BGDV Greece, S: NC_005233; M: NC_005234; L: NC_005235), Hantaan orthohantavirus (HTNV 76-118, S: NC_005218; M: NC_005219; L: NC_005222) and Seoul orthohantavirus (SEOV 80-39, S: NC_005236; M: NC_005237; L: NC_005238), and bat-borne hantaviruses, Brno loanvirus (BRNV 7/2012/CZE, S: KX845678; M: KX845679; L: KX845680) from *Nyctalus noctula*, Láibīn mobatvirus (LAIV BT20, S: KM102247; M: KM102248; L: KM102249) from *Taphozous melanopogon*, Xuân Sơn mobatvirus (XSV VN1982B4, S: KC688335; L: JX912953) from *Hipposideros pomona*, Quezon mobatvirus (QZNV MT1720/1657, S: KU950713; M: KU950714; L: KU950715) from *Rousettus amplexicaudatus*, Magboi virus (MGBV 1209, L: JN037851) from *Nycteris hispida*, Mouyassué virus (MOYV KB576, L: JQ28771) from *Neoromicia nanus*, Makokou virus (MAKV GB303, L: KT316176) from *Hipposideros ruber*, Huángpí virus (HUPV Pa-1, S: JX473273 and L: JX465369) from *Pipistrellus abramus*, Lóngquán loanvirus (LQUV Ra-25, S: JX465415; M: JX465397; and L: JX465381) from *Rhinolophus sinicus*, Đakrông mobatvirus (DKGV VN2913B72, S: MG663536; M: MG663535; L: MG663534) from *Aselliscus stoliczkanus*, respectively. The numbers at each node are posterior node probabilities based on 45,000 trees: two replicate Markov Chain Monte Carlo runs consisting of six chains of two million generations each sampled every 100 generations with a burn-in of 7500 (25%). The scale bar indicates the nt substitutions per site. The GenBank accession numbers for the S-, M- and L-segment sequences of soricine shrew-borne hantaviruses included in the analysis are provided in Supplementary Table S2.

Phylogenetic trees, based on the coding regions of the full-length S and M and partial L segments, were constructed by Bayesian methods. Seewis orthohantavirus sequences (Supplementary Table S2) from 79 Eurasian shrews, 10 Laxmann’s shrews (MSB148347, MSB148436, MSB148457, MSB148558, MSB148559, ART502, Parnaya-Sc1205, Telet-Sc170, Khekhtsir-Sc1126, Galkino-Sc2712) and 2 tundra shrews (Galkino-St48, Galkino-St2714) segregated into separate clades with their respective host species (Figure 2). However, SWSV from two tundra shrews (MG373S022, Irkutsk-St489) and one Siberian large-toothed shrew (Irkutsk-Sd475) clustered with SWSV from Eurasian shrews (Figure 2). 

The other highly divergent full-length and partial S-, M- and L-genomic sequences from 20 soricine shrews (four *S. araneus*, 12 *S. caecutiens*, two *S. roboratus*, one *S. tundrensis* and one *S. minutus*) formed two clades, ALTV and LENV, which shared a common ancestry with Camp Ripley virus (RPLV) strain MSB90845 from the northern short-tailed shrew (*Blarina brevicauda*) in the S and L trees (Figure 2) and with Quezon virus (QZNV) from the Geoffroy’s rousette (*Rousettus amplexicaudatus*) in the M tree. Based on the S- and L-segment phylogeny, ALTV strains from *S. araneus* in Hungary (MSB95363, MSB95469) and Finland (Uurainen63L, LohjaEWS10L) clustered with prototype ALTV ALT302 and a new strain Parabel-Sa44 from Western Siberia (Figure 2). LENV strains from *S. caecutiens* (Parnaya-Sc1217, Khekhtsir-Sc67, MSB146482, MSB148580, MSB148458, MSB148573, MSB148574, MSB148745, MSB148793) and *S. roboratus* (MSB148839, MSB148679) in Russia and from *S. tundrensis* (MG361S013) in Mongolia clustered with LENV strain from *S. minutus* found in Poland (Smin1108) and formed a second clade (Figure 2). 

Similarly, in the M-segment tree, hantavirus sequences from three *S. caecutiens* (MSB148580, MSB148458, MSB148793) and one *S. roboratus* (MSB148679) clustered with LENV strain Khekhtsir-Sc67 (Figure 2), while prototype strain ALTV ALT302 formed a separate lineage. In the L-segment tree, hantavirus from one *S. minutissimus* (Smi458 MSB1486651), captured in Russia, segregated with RPLV (MSB90845), from the United States. The overall topology of the S- and M-segment trees were more similar and suggested that ALTV and LENV belonged to the *Mobatvirus* genus. Geographic-specific clustering was evident for LENV strains from *S. caecutiens* and *S. roboratus* captured along Kenkeme River and Amga River (Figure 2).

**Figure 3:**
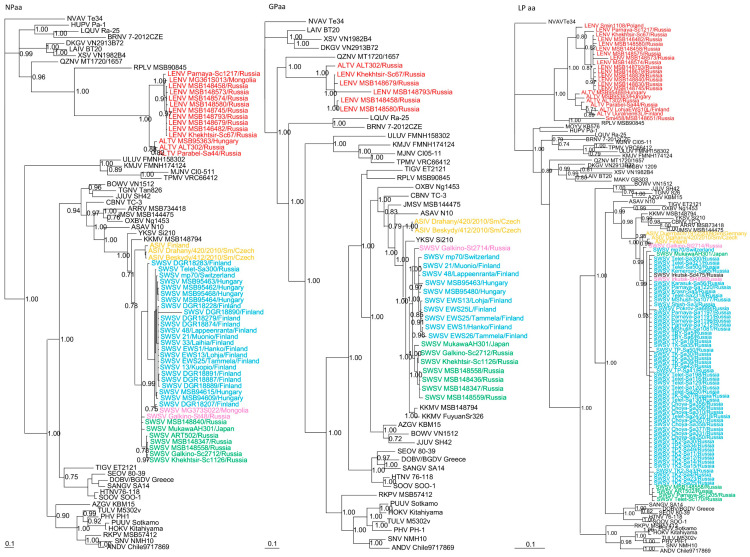
Phylogenetic trees generated by the Bayesian method, based on the nucleocapsid protein (NPaa), glycoprotein (GPaa) and RNA-dependent RNA polymerase (LPaa) of ALTV (red), SWSV strains from *Sorex araneus* (light blue), SWSV strains from *Sorex caecutiens* (green), SWSV strains from *Sorex tundrensis* (light pink) and Asikkala virus (ASIV) from *Sorex minutus* (yellow). The phylogenetic positions of other representative hantaviruses hosted by rodents, shrews, moles and bats are also shown. GenBank accession numbers are as shown in Figure 2 and in Supplementary Table S2.

Phylogenetic trees, based on complete N, GPC and partial RdRp protein sequences also revealed similar topologies with distinct clustering of SWSV and ALTV strains (Figure 3). SWSV strains from *S. araneus*, *S. tundrensis, S. caecutiens* and *S. daphaenodon* were more closely related to members of the genus *Orthohantavirus* and were associated with other *Sorex* species. Phylogeographic variation of SWSV strains reflected the host association, geography and distinct evolutionary histories of SWSV and ARTV variants [22,24,25]. ALTV from *S. araneus* and LENV from *S. caecutiens, S. roboratus, S. tundrensis* and *S. minutus* were more closely related to members of the genus *Mobatvirus*, associated mainly with bats, but also with mole (NVAV) and shrew (RPLV strain MSB90845), that have reassorted M and L genome segments [17]. Phylogeographic variation of LENV strains reflected the host associations and their geographic origins.

## 4. Discussion

Our data and previously published data demonstrate widespread circulation of SWSV throughout the geographic range of *S. araneus*, spanning across Central and Northern Europe to Eastern Siberia. SWSV was found in all new capture sites located in Western Siberia (Altai Republic and Tomsk Oblast). Moreover, in Russia and Mongolia, SWSV was also detected in other closely related *Sorex* species, such as the tundra shrew and Siberian large-toothed shrew [22]. The two distinct genetic variants of SWSV, previously named ARTV, were detected in *S. caecutiens* and *S. tundrensis*. ARTV in *S. caecutiens* was found across its geographic range in Asian Russia (Altai Republic, Sakha Republic, Krasnoyarsk and Khabarovsk Krai) and Japan, while ARTV in *S. tundrensis* was detected in far-eastern Russia (Khabarovsk Krai) [25]. 

In addition to SWSV, highly divergent hantaviruses, designated ALTV and LENV, were detected in *S. araneus* and *S. caecutiens*, *S. tundrensis*, and *S. roboratus*, respectively. Here, we report the genomic characterization of ALTV, a new hantavirus in *S. araneus*, previously recognized as the principal reservoir of SWSV. Our data demonstrate co-circulation of two divergent hantaviruses in the same host species and locations in Western Siberia. Finding of ALTV in *S. araneus* in two Siberian sites, as well as in Finland and Hungary [8,9] may indicate that *S. araneus* is the reservoir host of ALTV. However, the possibility that *S. araneus* may be a spillover host of ALTV cannot be completely rejected. This report and our previous studies demonstrated very low level of ALTV infection rate among *S. araneus* [22,33]. Since 2007, ALTV was detected in two of 258 samples from *S. araneus*, captured in Siberia, while SWSV sequences were detected in 59 samples. Initially ALTV, SWSV, and ARTV had been found in one locality near Teletskoye Lake in the Altai Republic. During this study ALTV was found in a distant locality of Tomsk Oblast. 

These data suggest two possible hypotheses. First, differential sensitivity of host (*S. araneus*) to ALTV and SWSV infection due to different evolution histories of these viruses. Long-term co-evolution of SWSV and its natural host provide better infection sensitivity for SWSV compared to ALTV, which suggest ancient host-switching event from another reservoir host during evolution. Second, differential interaction between host and virus for ALTV and SWSV. SWSV demonstrated persistent infection, while ALTV might have a short period of active infection and then complete elimination by the immune system of the host shrew. As a consequence, pathogenicity of ALTV for humans might be distinct compared to SWSV, which most probably is non-pathogenic for humans [37]. Both hypotheses warrant further investigation, including attempts to isolate SWSV and ALTV.

The full-length S-genomic segment of ALTV strain ALT 302 encoded an N protein of 448 aa, 12–24 aa longer than for other hantaviruses. The same length of N protein was found for LENV, supporting a common evolutionary origin. Additional aa were located at the N-terminus of the N protein. The first 110 aa showed the highest degree of diversity compared to other hantaviruses, more than 61%. As the N-terminal 107 aa contain immunodominant linear epitopes [38,39], we posit that available ELISA kits, based on the N proteins of Hantaan, Seoul and Puumala viruses, would not be able to detect ALTV or LENV infection in humans.

## 5. Conclusions

An ALTV-related hantavirus harbored by *S. caecutiens*, provisionally named LENV, might represent a distinct hantavirus species. LENV was detected in *S. caecutiens* and *S. roboratus*, which inhabit the same locality in Sakha Republic, as well as in *S. tundrensis* in Mongolia. LENV was detected throughout the eastern-most geographic range of its host, from Eastern Siberia (Krasnoyarsk Krai) up to far-eastern Russia (Sakha Republic, Khabarovsk Krai). LENV was also found in Poland, where a closely related strain was identified in *S. minutus*. The findings of LENV in multiple *Sorex* species from widely separated geographic regions indicate that several *Sorex* species may represent the reservoir hosts of genetic variants of LENV. Further, a highly divergent ALTV-related hantavirus from *S. minutissimus*, captured in Russia and phylogenetically more closely related to RPLV, found in *Blarina brevicauda* from the United States [17,40], probably represents a new hantavirus species.

## Figures and Tables

**Table 1:** Prevalence of hantavirus RNA and identified viruses in *Sorex* shrews by species and location in Asian Russia.

Region	Collection Site	Species	Year	Hantavirus RNA Positive/Tested	Virus
**Altai Republic**	**Teletskoye**	*Sorex araneus*	2007	7/10	SWSV, ALTV
		*Sorex caecutiens*	2007	1/1	ARTV
		***Sorex araneus***	**2018**	**1/6**	**SWSV**
		***Sorex araneus***	**2019**	**6/43**	**SWSV**
		***Sorex caecutiens***	**2019**	**1/1**	**ARTV**
		***Sorex minutus***	**2019**	**0/2**	**-**
	**Choya**	***Sorex araneus***	**2019**	**8/23**	**SWSV**
		***Sorex isodon***	**2019**	**0/1**	**-**
**Tomsk Oblast**	**Belyi Yar**	***Sorex araneus***	**2019**	**2/10**	**SWSV**
		***Sorex caecutiens***	**2019**	**0/3**	**-**
	**Volkovo**	***Sorex araneus***	**2019**	**1/7**	**SWSV**
	**Kargasok**	***Sorex araneus***	**2019**	**15/46**	**SWSV**
		***Sorex caecutiens***	**2019**	**0/1**	**-**
	**Parabel**	***Sorex araneus***	**2019**	**6/28**	**SWSV, ALTV**
		***Sorex caecutiens***	**2019**	**0/2**	**-**
Khabarovsk Krai	Khekhtsir	*Sorex caecutiens*	2007	1/13	ARTV
		*Sorex caecutiens*	2008	1/7	LENV
	Galkino	*Sorex caecutiens*	2007	2/28	ARTV
		*Sorex tundrensis*	2007	10/32	ARTV
		*Sorex caecutiens*	2008	0/11	-
		*Sorex tundrensis*	2008	4/13	ARTV
Krasnoyarsk Krai	Parnaya	*Sorex araneus*	2008	5/17	SWSV
		*Sorex caecutiens*	2008	2/2	ARTV, LENV
		*Sorex tundrensis*	2008	0/3	-
Irkutsk Oblast	Irkutsk City	*Sorex araneus*	2007	0/2	-
		*Sorex tundrensis*	2007	1/2	SWSV
		*Sorex daphaenodon*	2007	1/2	SWSV
**Sakha Republic**	**Amga River**	***Sorex caecutiens***	**2006**	**10/19**	**ARTV, LENV**
		***Sorex minutissimus***	**2006**	**1/5**	**ALTV-like**
	**Kenkeme River**	***Sorex caecutiens***	**2006**	**4/24**	**LENV**
		***Sorex roboratus***	**2006**	**4/12**	**LENV**
		***Sorex daphaenodon***	**2006**	**0/4**	**-**
	**Lena River**	***Sorex caecutiens***	**2006**	**1/6**	**LENV**
		***Sorex tundrensis***	**2006**	**0/5**	**-**

Samples in the present study are shown in bold font.

## Data Availability

The data about shrews and trap sites presented in this study are openly available in Arctos (http://arctos.database.museum/), an online collection management information system and provider of research-grade data. GenBank accession numbers for sequence data are available in the legend of Figure 2 and in Supplemental Table S2. Other data presented in this study are available on request from the corresponding authors.

## Supplementary Materials

The following are available online at https://www.mdpi.com/article/10.3390/v13071286/s1. Table S1: Oligonucleotide primers for amplification of the S, M and L segments of soricine shrew-borne hantaviruses, Table S2: GenBank accession numbers and host information for soricine shrew-borne hantaviruses included in genetic and phylogenetic analysis.

## Nucleotide Sequences

GenBank accession numbers are shown in Supplementary Table S2.
