# Supplementary material for: Geographic Distribution and Phylogeny of Soricine Shrew-Borne Seewis Virus and Altai Virus in Russia
Source: Viruses. 2021 Jul 1;13(7):1286. doi: 10.3390/v13071286 (PMC8310073; doi:10.3390/v13071286)
Supplement: Supplementary file 1 [file viruses-13-01286-s001.zip › viruses-1272087-supplementary.pdf]

viruses

MDPI

Supplementary Information

Geographic Distribution and Phylogeny of Soricine Shrew-Borne Seewis Virus and Altai Virus in Russia

Liudmila N. Yashina [1,*], Sergey A. Abramov [2], Alexander V. Zhigalin [3], Natalia A. Smetannikova [1], Tamara A. Dupal [2], Anton V. Krivopalov [2], Fuka Kikuchi [4,5], Kae Senoo [5,6], Satoru Arai [5], Tetsuya Mizutani [4], Motoi Suzuki [5], Joseph A. Cook [7] and Richard Yanagihara [8,*]

[1] State Research Center of Virology and Biotechnology, Vector, Koltsovo 630559, Russia; smetannikova@vector.nsc.ru
[2] Institute of Systematics and Ecology of Animals, Novosibirsk 630091, Russia; terio@eco.nsc.ru (S.A.A.); dupalgf54@gmail.com (T.A.D.); krivopalov@gmail.com (A.V.K.)
[3] Department of Vertebrate Zoology and Ecology, Tomsk State University, Tomsk 634050, Russia; alex-zhigalin@mail.ru
[4] Center for Infectious Disease Epidemiology and Prevention Research, Tokyo University of Agriculture and Technology, Tokyo 183-8538, Japan; S203828z@st.go.tuat.ac.jp (F.K.); tmizutan@cc.tuat.ac.jp (T.M.)
[5] Center for Surveillance, Immunization and Epidemiologic Research, National Institute of Infectious Diseases, Tokyo 162-8640, Japan; arais@nih.go.jp (S.A.); mosuzuki@niid.go.jp (M.S.)
[6] Faculty of Science, Tokyo University of Science, Tokyo 162-8601, Japan; 2319061@ed.tus.ac.jp
[7] Department of Biology and Museum of Southwestern Biology, University of New Mexico, Albuquerque, NM 87131, USA; cookjose@unm.edu
[8] Department of Pediatrics, John A. Burns School of Medicine, University of Hawaii at Manoa, Honolulu, HI 96813, USA
* Correspondence: yashina@vector.nsc.ru (L.N.Y.); ryanagih@hawaii.edu (R.Y.)

Citation: Yashina, L.N.; Abramov, S.A.; Zhigalin, A.V.; Smetannikova, N.A.; Dupal, T.A.; Krivopalov, A.V.; Kikuchi, F.; Senoo, K.; Arai, S.; Mizutani, T.; et al. Geographic Distribution and Phylogeny of Soricine Shrew-Borne Seewis Virus and Altai Virus in Russia. *Viruses* **2021**, *13*, 1286. https://doi.org/10.3390/v13071286

Academic Editors: Kumiko Yoshimatsu, Hiroaki Kariwa

Received: 8 June 2021
Accepted: 28 June 2021
Published: 1 July 2021

Publisher's Note: MDPI stays neutral with regard to jurisdictional claims in published maps and institutional affiliations.

Copyright: © 2021 by the authors. Licensee MDPI, Basel, Switzerland. This article is an open access article distributed under the terms and conditions of the Creative Commons Attribution (CC BY) license (http://creativecommons.org/licenses/by/4.0/).

Table S1. Oligonucleotide primers used to amplify the S, M and L segments of soricine shrew-borne hantaviruses.

Segment	Primer	Sequence (5`-3`) (Polarity)
SML	OSM55	TAG TAG TAG ACT CC (+/-)
S	S2FR	TAG TAG TAK RCT CCC TAA ARA G (+/-)
	4	CCD GGT GTW AKY TCT TCW GC (-)
	SAF1	GGA GCA YAA AGG RAA TAG GA (+)
	SAF2	GGT GAT GAC ATG GAT GCT AAC (+)
	S2	AGC TCA GGA TCC ATG TCA TC (-)
	SA3R	CCA ATC WGC WGT YTG YCC AC (-)
M	M5	TAG TAG TAG GAT CCG CAA GAA (+/-)
	MA600R	CTT AGC YTG ATA CAT KGG YTG A (-)
	MA460F	GAT CTG CCA GAC AGT CAG AAG (+)
	MA800R	CAA GGG AGT AGC CTT GGA AGA AG (-)
	MA1195R	CCT GTC GGY GAR AAG GCC TCA C (-)
	MA1490F	TGT GTN CCW GGN TTY CAT GG (+)
	M1190F	GGN CCN GGD GCW NVH TGT GA (+)
	M1500R	CCC AAC CGT GWA CAC CWG TRA CAC (-)
	M2237F	TTT CAY TGY TAT GGT GCD TG (+)
	MA2300R	GGA TTG CAT GCC CAG CCA CTC TGG (-)
	M1990R	ACW CCA TGT GCT GTA TCA TAC C (-)
	Han-M2631R	CAT NAY RTC NCC RGG RTC NCC (-)
	M2620F	GGA GAC ACA TTG GTT TTC CTT GGC (+)
	M3ER	TAG TAG TAG ACT CCG CAA GAA (-)
	MA3300F	GGT AAC TGG TGG GTM ATC C (+)
L	L5-1	TTC TGC AGT AGT AGT AGA CTC CG (+)
	LH2	ACA CCA TCA TTY CTY STA CTK GGC CA (-)
	RPLV-L-366F	RGT CAC TGT GAC AGY WGA TGT (+)
	SL-1458R	AKT AIA TGS CCT ATA TGC CAT GC (-)
	LA5170F	GGR AAR CAR TAT GAT GCH TAT TG (+)
	LA6070R	GTA TAA GCA CTC TCA TCY TGC (-)
	PHL-2111F	CAG TCW ACA RTT GGT GCA AGT GG
	HTL-2971R	CWG GWG ACC AYT TIG TDG CAT C (-)
	LA6000F	CAG GAT GAR AGT GCT TAT ACT RC (+)
	LA6440R	ATA AAA GGA AGA GGA CCT RTC (-)
	L3R	TAG TAG TAK GCT CCG CTG CAG (-)
	L1382F	AGA AGG TAG GAC TTA TGC AGA CAC (+)
	L2215R	CCA YGM AGV CCY TTY TCA AA (-)
	Han-L3588R	GGN ATH GAN CAN GCA CAN CCY TCA AA (-)
	L5178R	CAA TAN GCA TCA TAY TGY TTN CC (-)
	PHL-3endR	GAT WAA GCA TGA YTG GTC TGA (-)
	HAN-L-F1	ATG TAY GTB AGT GCW GAT GC (+)
	HAN-L-R1	AAC CAD TCW GTY CCR TCA TC (-)
	HAN-L-F2	TGC WGA TGC HAC NAA RTG GTC (+)
	HAN-L-R2	GCR TCR TCW GAR TGR TGD GCA A (-)
	RPLV-L-4837R	CYG TMC CYT CWA CAT TAC CTT (-)
	L3258F	TCC CTT TAT AGT GTG GGT GTG A (+)
	L6388R	CTC WGT YAA RTC ATA WGG ATC (-)
	L4091F	AAA AGT GAT TTT GAA TTT CAT GAT A (+)
	L4718F	CCA GAG GTT ATG CGT AGG GTA (+)
	PHL-5167R	CAT AYT GYT THC CTG AAT AWG C (-)
	RPLV-L-4837R	CYG TMC CYT CWA CAT TAC CTT G (-)

Abbreviations: A, Adenine; B, C or G or T; C, Cytosine; D, A or G or T; G, Guanine; H, A or C or T; I, Inosine; K, G or T; M, A or C; N, any nucleotide; R, A or G; S, G or C; T, Thymine; V, A or C or G; W, A or T; Y, C or T.

Table S2. GenBank accession numbers and host information for soricine shrew-borne hantaviruses included in genetic and phylogenetic analysis.

Virus Name	Strain Name	Country	Location	Host Species	Sex	Capture Date		S Segment Accession no.	M Segment Accession no.	L Segment Accession no.
						Month	Year			
Lena River virus	MSB148458	Russia	Amga River	*Sorex caecutiens*	male	August	2006	KM361045	KM361052	KM361057
Lena River virus	MSB148573	Russia	Amga River	*Sorex caecutiens*	male	August	2006	KM361046	-	KM361058
Lena River virus	MSB148574	Russia	Amga River	*Sorex caecutiens*	male	August	2006	KM361047	-	KM361059
Lena River virus	MSB148575	Russia	Amga River	*Sorex caecutiens*	male	August	2006	-	-	MN401400
Lena River virus	MSB148580	Russia	Amga River	*Sorex caecutiens*	male	August	2006	KM361044	KM361051	KM361056
Lena River virus	Smin1108	Poland	Chmiel	*Sorex minutus*	female	September	2010	-	-	MN244248
Lena River virus	MSB148745	Russia	Kenkeme River	*Sorex caecutiens*	male	August	2006	KM361050	-	KM361060
Lena River virus	MSB148793	Russia	Kenkeme River	*Sorex caecutiens*	female	August	2006	KM361048	KM361053	KM361061
Lena River virus	MSB148830	Russia	Kenkeme River	*Sorex caecutiens*	male	August	2006	-	-	MN401398
Lena River virus	MSB148840	Russia	Kenkeme River	*Sorex caecutiens*	male	August	2006	MT951407	-	MN401399
Lena River virus	MSB148679	Russia	Kenkeme River	*Sorex roboratus*	male	August	2006	KM361049	KM361054	KM361063
Lena River virus	MSB148839	Russia	Kenkeme River	*Sorex roboratus*	female	August	2006	-	-	KM361062
Lena River virus	MSB146482	Russia	Lena River	*Sorex caecutiens*	male	August	2010	KM361043	-	KM361055
Lena River virus	Khekhtsir-Sc67	Russia	Khekhtsir	*Sorex caecutiens*	female	February	2008	MH499470	MH499471	MH499472
Lena River virus	Parnaya-Sc1217	Russia	Parnaya	*Sorex caecutiens*	male	August	2008	MW505551	-	MW505552
Lena River virus	MG361S013	Mongolia	Hovsgol Lake	*Sorex tundrensis*	male	August	2010	MT544323	-	-
Altai virus	MSB95363	Hungary	Nógrád	*Sorex araneus*	male	July	1997	GQ293127	-	GQ293104
Altai virus	MSB95469	Hungary	Gyor-Sopron-Moson	*Sorex araneus*		July	1997	-	-	GQ293105
Altai virus	ALT302	Russia	Teletskoye Lake	*Sorex araneus*	female	August	2007	MK340902	MK340903	MT648514
Altai virus	Parabel-Sa44	Russia	Parabel	*Sorex araneus*	male	May	2019	MT560057	-	MN815789
Altai virus	Uurainen63L	Finland	Uurainen	*Sorex araneus*		October	2004	-	-	KJ136623
Altai virus	LohjaEWS10L	Finland	Lohja	*Sorex araneus*		November	2012	-	-	KJ136638
Altai-like virus	MSB148651	Russia	Amga River	*Sorex minutissimus*	male	August	2006	-	-	MN244247
Seewis orthohantavirus	MSB148347	Russia	Amga River	*Sorex caecutiens*	male	August	2006	KM20149	KM201420	KM201421
Seewis orthohantavirus	MSB148436	Russia	Amga River	*Sorex caecutiens*	male	August	2006	-	KM201415	KM201416

Seewis ortho-hantavirus	MSB148457	Russia	Amga River	*Sorex caecutiens*		August	2006	-	KM201417	KM201418
Seewis ortho-hantavirus	MSB148558	Russia	Amga River	*Sorex caecutiens*	male	August	2006	KM201411	KM201412	KM20143
Seewis ortho-hantavirus	MSB148559	Russia	Amga River	*Sorex caecutiens*	female	August	2006		MT951408	KM201414
Seewis ortho-hantavirus	ART502	Russia	Teletskoye Lake	*Sorex caecutiens*	female	September	2007	MN815798	EU424340	EU424339
Seewis ortho-hantavirus	Parnaya-Sc1205	Russia	Parnaya	*Sorex caecutiens*	female	August	2008	KU253274	-	KU253275
Seewis ortho-hantavirus	Telet-Sc170	Russia	Teletskoye Lale	*Sorex caecutiens*	male	August	2019	-	-	MN815794
Seewis ortho-hantavirus	Galkino-Sc2712	Russia	Galkino	*Sorex caecutiens*	female	Novem-ber	2007	MG888404	MG913807	MG860918
Seewis ortho-hantavirus	Khekhtsir-Sc1126	Russia	Khekhtsir	*Sorex caecutiens*	female	July	2007	MG888405	MG913808	MG860919
Seewis ortho-hantavirus	DGR18207	Finland	Lappi	*Sorex araneus*	female	August	1982	GQ293126	-	GQ293103
Seewis ortho-hantavirus	DGR18228	Finland	Hame	*Sorex araneus*	male	August	1982	GQ293125	-	GQ293101
Seewis ortho-hantavirus	DGR18283	Finland	Hame	*Sorex araneus*	female	August	1982	GU186445	-	GQ293100
Seewis ortho-hantavirus	DGR18279	Finland	Hame	*Sorex araneus*	female	August	1982	GQ293131	-	GQ293110
Seewis ortho-hantavirus	DGR18874	Finland	Oulun Lääni	*Sorex araneus*	female	August	1982	GQ293132	-	GQ293111
Seewis ortho-hantavirus	DGR18887	Finland	Oulun Lääni	*Sorex araneus*	female	August	1982	GQ293133	-	GQ293112
Seewis ortho-hantavirus	DGR18889	Finland	Oulun Lääni	*Sorex araneus*	male	August	1982	GQ293134	-	GQ293114
Seewis ortho-hantavirus	DGR18890	Finland	Oulun Lääni	*Sorex araneus*	male	August	1982	GQ293130	-	GQ293109
Seewis ortho-hantavirus	DGR18891	Finland	Oulun Lääni	*Sorex araneus*	female	August	1982	GQ293129	-	GQ293108
Seewis ortho-hantavirus	MSB95461	Hun-gary	Gyor-Sopron-Moson	*Sorex araneus*	female	July	1997	-	-	GQ293098
Seewis ortho-hantavirus	MSB95462	Hun-gary	Gyor-Sopron-Moson	*Sorex araneus*	female	July	1997	GQ293124	-	GQ293097
Seewis ortho-hantavirus	MSB95463	Hun-gary	Gyor-Sopron-Moson	*Sorex araneus*	female	July	1997	GQ293136	GQ293140	GQ293116
Seewis ortho-hantavirus	MSB95464	Hun-gary	Gyor-Sopron-Moson	*Sorex araneus*	female	July	1997	GQ293137		GQ293117
Seewis ortho-hantavirus	MSB95468	Hun-gary	Gyor-Sopron-Moson	*Sorex araneus*	female	July	1997	GQ293128	-	GQ293106
Seewis ortho-hantavirus	MSB95480	Hun-gary	Gyor-Sopron-Moson	*Sorex araneus*		July	1997	-	GQ293139	GQ293121
Seewis ortho-hantavirus	MSB94609	Hun-gary	Zala	*Sorex araneus*	male	June	2000	GQ293138	-	GQ293123
Seewis ortho-hantavirus	MSB94615	Hun-gary	Zala	*Sorex araneus*		June	2000	GQ293135	-	GQ293115
Seewis ortho-hantavirus	Kemerovo-Sa65	Russia	Kemerovo	*Sorex araneus*	male	July	2008	GQ284582	-	GQ267812
Seewis ortho-hantavirus	Karasuk-Sa56	Russia	Karasuk	*Sorex araneus*	male	June	2008	GQ284586	-	GQ267809
Seewis ortho-hantavirus	Krasn-Sa5	Russia	Krasnoyarsk	*Sorex araneus*		August	2008	GQ284584	-	GQ267811

Seewis orthohantavirus	Shish-Sa3	Russia	Shish	*Sorex araneus*		June	2017		-	MK005198
Seewis orthohantavirus	Pokrov-Sa689	Russia	Pokrovka	*Sorex araneus*	female	June	2008	-	-	MK005199
Seewis orthohantavirus	Parnaya-Sa1191	Russia	Parnaya	*Sorex araneus*	female	August	2008	-	-	MG279208
Seewis orthohantavirus	Parnaya-Sa1196	Russia	Parnaya	*Sorex araneus*	male	August	2008	-	-	MG279209
Seewis orthohantavirus	Parnaya-Sa1197	Russia	Parnaya	*Sorex araneus*	female	August	2008	MG279214	-	MG279210
Seewis orthohantavirus	Parnaya-Sa1212	Russia	Parnaya	*Sorex araneus*	male	August	2008	MG279215	-	MG279211
Seewis orthohantavirus	Parnaya-Sa1220	Russia	Parnaya	*Sorex araneus*	male	August	2008	GQ284580	-	GQ267810
Seewis orthohantavirus	MShush-Sa1077	Russia	Middle Shush	*Sorex araneus*	male	August	2008	MG279216	-	MG279212
Seewis orthohantavirus	MShush-Sa1081	Russia	Middle Shush	*Sorex araneus*	female	August	2008	MG279217	-	MG279213
Seewis orthohantavirus	TBY-Sa5	Russia	Belyi Yar	*Sorex araneus*	male	April	2019	-	-	MT560032
Seewis orthohantavirus	TBY-Sa6	Russia	Belyi Yar	*Sorex araneus*	male	April	2019	-	-	MT560033
Seewis orthohantavirus	TV-Sa18	Russia	Volkovo	*Sorex araneus*	female	April	2019	-	-	MT560034
Seewis orthohantavirus	TK2-Sa3	Russia	Kargasok	*Sorex araneus*	male	August	2019	-	-	MT560037
Seewis orthohantavirus	TK2-Sa4	Russia	Kargasok	*Sorex araneus*	female	August	2019	-	-	MT560038
Seewis orthohantavirus	TK2-Sa11	Russia	Kargasok	*Sorex araneus*	male	August	2019	-	-	MT560039
Seewis orthohantavirus	TK2-Sa12	Russia	Kargasok	*Sorex araneus*	female	August	2019	-	-	MT560040
Seewis orthohantavirus	TK2-Sa14	Russia	Kargasok	*Sorex araneus*	female	August	2019	-	-	MT560041
Seewis orthohantavirus	TK2-Sa15	Russia	Kargasok	*Sorex araneus*	female	August	2019	-	-	MT560042
Seewis orthohantavirus	TK2-Sa23	Russia	Kargasok	*Sorex araneus*	male	August	2019	-	-	MT560043
Seewis orthohantavirus	TK2-Sa25	Russia	Kargasok	*Sorex araneus*	female	August	2019	-	-	MT560044
Seewis orthohantavirus	TK2-Sa30	Russia	Kargasok	*Sorex araneus*	male	August	2019	-	-	MT560045
Seewis orthohantavirus	TK-Sa30	Russia	Kargasok	*Sorex araneus*	male	May	2019	-	-	MT560035
Seewis orthohantavirus	TK-Sa33	Russia	Kargasok	*Sorex araneus*	female	May	2019	-	-	MT560036
Seewis orthohantavirus	TK-Sa35	Russia	Kargasok	*Sorex araneus*	female	May	2019	-	-	MN815783
Seewis orthohantavirus	TK-Sa37	Russia	Kargasok	*Sorex araneus*	male	August	2019	-	-	MN815784
Seewis orthohantavirus	TK-Sa39	Russia	Kargasok	*Sorex araneus*	male	May	2019	-	-	MN815785
Seewis orthohantavirus	TP2-Sa34	Russia	Parabel	*Sorex araneus*	female	August	2019	-	-	MT560046
Seewis orthohantavirus	TP2-Sa49	Russia	Parabel	*Sorex araneus*	male	August	2019	-	-	MT560047

Seewis orthohantavirus	TP-Sa42	Russia	Parabel	*Sorex araneus*	male	May	2019	-	-	MN815787
Seewis orthohantavirus	TP-Sa41	Russia	Parabel	*Sorex araneus*	male	May	2019	-	-	MN815786
Seewis orthohantavirus	TP-Sa50	Russia	Parabel	*Sorex araneus*	female	May	2019	-	-	MN815788
Seewis orthohantavirus	Telet-Sa24	Russia	Teletskoye Lake	*Sorex araneus*	male	June	2018	-	-	MT560048
Seewis orthohantavirus	Telet-Sa116	Russia	Teletskoye Lake	*Sorex araneus*	female	August	2019	-	-	MN815790
Seewis orthohantavirus	Telet-Sa120	Russia	Teletskoye Lake	*Sorex araneus*	male	August	2019	-	-	MN815791
Seewis orthohantavirus	Telet-Sa129	Russia	Teletskoye Lake	*Sorex araneus*	male	August	2019	-	-	MN815792
Seewis orthohantavirus	Telet-Sa130	Russia	Teletskoye Lake	*Sorex araneus*	male	August	2019	-	-	MN815793
Seewis orthohantavirus	Telet-Sa198	Russia	Teletskoye Lake	*Sorex araneus*	female	August	2019	-	-	MN815795
Seewis orthohantavirus	Telet-Sa250	Russia	Teletskoye Lake	*Sorex araneus*	male	August	2019	-	-	MN815796
Seewis orthohantavirus	Telet-Sa300	Russia	Teletskoye Lake	*Sorex araneus*	female	August	2007	MN815797	-	EU424334
Seewis orthohantavirus	Telet-Sa321	Russia	Teletskoye Lake	*Sorex araneus*	male	August	2007	GQ284575	-	EU424337
Seewis orthohantavirus	Telet-Sa500	Russia	Teletskoye Lake	*Sorex araneus*	female	September	2007	GQ284576	-	EU424338
Seewis orthohantavirus	Telet-Sa2318	Russia	Teletskoye Lake	*Sorex araneus*	male	October	2009	-	-	MK005197
Seewis orthohantavirus	Choya-Sa310	Russia	Choya	*Sorex araneus*	female	August	2019	-	-	MT560049
Seewis orthohantavirus	Choya-Sa331	Russia	Choya	*Sorex araneus*	female	August	2019	-	-	MT560050
Seewis orthohantavirus	Choya-Sa350	Russia	Choya	*Sorex araneus*	female	August	2019	-	-	MT560051
Seewis orthohantavirus	Choya-Sa356	Russia	Choya	*Sorex araneus*	female	August	2019	-	-	MT560052
Seewis orthohantavirus	Choya-Sa357	Russia	Choya	*Sorex araneus*	female	August	2019	-	-	MT560053
Seewis orthohantavirus	Choya-Sa358	Russia	Choya	*Sorex araneus*	male	August	2019	-	-	MT560054
Seewis orthohantavirus	Choya-Sa376	Russia	Choya	*Sorex araneus*	male	August	2019	-	-	MT560055
Seewis orthohantavirus	Choya-Sa377	Russia	Choya	*Sorex araneus*	male	August	2019	-	-	MT560056
Seewis orthohantavirus	48/Lappeenranta	Finland	Lappeenranta	*Sorex araneus*		August	2010	KJ136609	KJ136603	-
Seewis orthohantavirus	21/Muonio/2009	Finland	Muonio	*Sorex araneus*		June	2009	KJ136610	KJ136604	-
Seewis orthohantavirus	33/Laihia/2005	Finland	Laihia	*Sorex araneus*		October	2005	KJ136611	-	-
Seewis orthohantavirus	EWS1/Hanko	Finland	Hanko	*Sorex araneus*		October	2012	KJ136612	KJ136607	-
Seewis orthohantavirus	EWS13/Lohja	Finland	Lohja	*Sorex araneus*		November	2012	KJ136613	KJ136605	-
Seewis orthohantavirus	EWS25/Tammela	Finland	Tammela	*Sorex araneus*		August	2012	KJ136614	KJ136608	-

Seewis ortho-hantavirus	13/Kuopio	Finland	Kuopio	*Sorex araneus*		August	2010	KJ136615	-	-
Seewis ortho-hantavirus	EWS26/Tam-mela	Finland	Tammela	*Sorex araneus*		August	2012	-	KJ136606	-
Seewis ortho-hantavirus	EWS25L	Finland	-	*Sorex araneus*		August	2012	-	-	KJ136634
Seewis ortho-hantavirus	Irkutsk-Sd475	Russia	Irkutsk	*Sorex daphaenodon*	female	June	2007	GQ284573	-	GQ267806
Seewis ortho-hantavirus	Irkutsk-St489	Russia	Irkutsk	*Sorex tundrensis*	male	June	2007	GQ284574	-	GQ267807
Seewis ortho-hantavirus	Galkino-St2714	Russia	Galkino	*Sorex tundrensis*	female	Novem-ber	2007	MG888402	MG913806	MH499473
Seewis ortho-hantavirus	Galkino-St48	Russia	Galkino	*Sorex tundrensis*	female	February	2008	MG888403	-	MG888365
Seewis ortho-hantavirus	MG373S022	Mongo-lia	Hovsgol Lake	*Sorex tundrensis*	male	August	2010	MT544324	-	-
Asikkala ortho-hantavirus	-	Finland	-	*Sorex minutus*		October	2005	KJ136616	-	KJ136628
Asikkala ortho-hantavirus	CZ/Beskydy/412/2010/Sm	Czech Republic	Beskydy	*Sorex minutus*			2010	KC880341	KC880344	KC880347
Asikkala ortho-hantavirus	CZ/Dra-hany/420/2010/Sm	Czech Republic	Drahany	*Sorex minutus*			2010	KC880342	KC880345	KC880348
Asikkala ortho-hantavirus	DE/Duerrbach/1912/2009/Sm	Ger-many	Duerrbach	*Sorex minutus*			2009	KC880343	KC880346	KC880349
Kenkeme or-thohantavirus	MSB148794	Russia	Kenkeme River	*Sorex roboratus*	male	August	2006	GQ306148	GQ306149	GQ306150
Boginia virus	LaihiaL	Finland	Laihia	*Neomys fodiens*		October	2005	-	-	KJ136642
